# Evolution and Circulation of Type-2 Vaccine-Derived Polioviruses in Nad Ali District of Southern Afghanistan during June 2009-February 2011

**DOI:** 10.1371/journal.pone.0088442

**Published:** 2014-02-18

**Authors:** Salmaan Sharif, Bilal Haider Abbasi, Adnan Khurshid, Muhammad Masroor Alam, Shahzad Shaukat, Mehar Angez, Muhammad Suleman Rana, Syed Sohail Zahoor Zaidi

**Affiliations:** 1 Department of Virology, National Institute of Health, Islamabad, Pakistan; 2 Department of Biotechnology, Quaid-e-Azam University, Islamabad, Pakistan; Columbia University, United States of America

## Abstract

Oral polio vaccine has been used successfully as a powerful tool to control the spread of wild polioviruses throughout the world; however, during replication in under immunized children, some vaccine viruses revert and acquire the neurovirulent phenotypic properties. In this study, we describe the evolution and circulation of Vaccine-Derived Polioviruses (VDPVs) in Helmand province of Afghanistan. We investigated 2646 AFP cases of Afghan children from June 2009–February 2011 and isolated 103 (04%) vaccine viruses, 45(1.7%) wild type polioviruses and six (0.22%) type 2 circulating vaccine-derived polioviruses (cVDPVs). These cVDPVs showed 97.7%–98.2% nucleotide and 98%–98.7% amino acid homology in VP1 region on comparison with Sabin type 2 reference strain. All these cVDPVs had two signature mutations of neurovirulent phenotypes and 12 additional mutations in P1 capsid region that might also have contributed to increase neurovirulence and replication. Phylogenetic analysis revealed that all these viruses were closely related and originated from previously reported Sabin like 2 virus from Pakistan which did not conform to the standard definition of VDPVs at that time. It was also observed that initial OPV dose was administered approximately 9 months prior to the collection of first stool specimen of index case. Our findings support that suboptimal surveillance and low routine immunization coverage have contributed to the emergence and spread of these viruses in Afghanistan. We therefore recommend high quality immunization campaigns not only in affected district Nad Ali but also in the bordering areas between Pakistan and Afghanistan to prevent the spread of cVDPVs.

## Introduction

Poliovirus (PV), the causative agent of acute paralytic disease poliomyelitis, is a member of enterovirus genus of *Picornaviridae* family and has three distinct serotypes (PV1, PV2 and PV3) [1]. It contains a positive sense 7.5 kb long single stranded RNA genome enclosed by icosahedral capsid which is made of four structural viral proteins VP1, VP2, VP3 and VP4. In addition, its genome also contains non-structural viral proteins (2A, 2B, 2C, 3A, 3B, 3C and 3D), 5′ and 3′ un-translated regions (UTRs) and a poly A tail [1].

For over five decades, poliomyelitis has been efficiently controlled by a live attenuated oral polio vaccine (OPV) which is amongst the best known viral vaccines in practice albeit with two disadvantages. Firstly, it can cause paralysis in extremely rare cases (approximately 1 per 2.7 million first doses of vaccine). Secondly, OPV viruses are genetically unstable [2] and vaccine strains may revert to pathogenic phenotypes during replication in human gut due to high error frequency during RNA synthesis (approximately 10^−4^ per base pair per replication cycle) [3]. These substitutions accumulate at an approximate rate of 1% per annum in viral protein 1 (VP1) region of genome [4] and can be used for estimation of evolutionary intervals between poliovirus isolates.

According to World Health Organization (WHO) recommendations, these OPV-derived reverent strains with 1% or more nucleotide mutations in the VP1 region are defined as Vaccine-Derived Polioviruses (VDPVs) [5]. In 2011, this criterion was modified down to >0.6% for only type2 VDPVs [6], [7].VDPVs have been classified into two well-defined categories known as Immunodeficiency associated VDPVs (iVDPVs) and Circulating VDPVs (cVDPVs) [7]. iVDPVs have been isolated from immune deficient patients in the United Kingdom (2000), United States (2008) [8], [9], Argentina (2009), Iran (2011), South Africa (2011) and China (2012) [10]. The second type, cVDPVs, arises as a result of sustained person to person transmission within an inadequately immunized population and first outbreak of these VDPVs was reported from Hispaniola (Haiti and the Dominican Republic) in 2001 [11]. Later on, these cVDPVs have been isolated in Egypt, Philippines, China, Madagascar, Nigeria, Myanmar, Indonesia, Cambodia, Somalia, Yemen, Niger, DRC and the United States [10]. Recently, a third category named as ambiguous VDPVs (aVDPVs) has emerged, which includes all those VDPVs whose ultimate source or host remained unidentified [12]. This type of polioviruses have been detected in Peru (1983 and 2011), Israel (1998 and 2006), Pakistan (2000), Romania (2002), Laos (2004), Madagascar (2005), Minnesota (2005), Myanmar (2006), Nigeria (2002, 2006 and 2012), Argentina (2011), DRC (2011), Finland (2011), India(2011), Sudan (2012) and Yemen (2012) [13], [14].

In October 1999, last wild poliovirus type 2 (WPV2) was isolated in Aligarh, Uttar Pradesh, India [15]. Since then, no WPV2 has been reported globally, however the largest outbreak of type 2 cVDPVs was recorded in Nigeria from 2005 to 2009 [16]. This outbreak provided a unique opportunity to analyze the pathogenicity of the virus, its associated disease severity, and the effectiveness of control measures to stop the spread of cVDPVs [17]. In this report, we describe the characterization of six cVDPV2 strains isolated from Acute Flaccid Paralysis (AFP) cases from Nad Ali district of Helmand province in Southern Afghanistan that remained in circulation for at least one year (2010 to 2011).

## Materials and Methods

### Sample Collection

Two stool samples were collected at 24 hours interval from suspected AFP cases within 14 days of paralysis onset and sent to WHO Regional Reference Laboratory for Polio Eradication Initiative (WHO RRL for PEI) at National institute of Health (NIH) Islamabad, Pakistan through WHO Afghanistan coordinated effort. The study concept and design was approved through the Pakistan's National Institute of Health Internal Review Board. The samples were collected after informed and written consent from the patient's parents/guardians. The samples were received within 72 hours after the collection of second specimen maintaining reverse cold chain.

### Virus Isolation and Characterization

The samples were processed according to WHO Polio laboratory protocol and clarified supernatant from each sample was inoculated on RD and L20B cell lines [18]. All L20B-positive isolates were subjected to real time reverse transcriptase polymerase chain reaction (rRT-PCR) for PV serotyping, intratypic differentiation (ITD) and primary screening for suspected VDPVs using WHO protocol [19]. Suspected VDPVs were referred for nucleotide sequencing of VP1 gene encoding structural protein region for final confirmation.

### Nucleic Acid sequencing

Viral RNA extraction, reverse transcriptase-PCR amplification and cycle sequencing of P1 capsid and 5′UTR were performed as described previously [20]–[22], using primers listed in Table 1. Bi-directional sequencing was performed using Big Dye Terminator kit v3.1 (Applied Biosystems) and each nucleotide was sequenced at least once from each strand.

**Table 1:** Sequence and position of Primer pairs used for PCR and Sequencing.

Primer Name	Primer Sequence	Polarity	Region
5′ UTR	TTAAAACAGCTCTGGGGTTG	Sense	5′UTR
241c	TACTGGGCTTTTCGAAGTAC	Antisense	5′UTR
UG53	TGGCTGCTTATGGTGACAAT	Sense	5′UTR
921c	CGTCCTTAATGGGTTCGGTG	Antisense	5′UTR
1737	GTGTWGTGAGTTCAA[Y]GG	Sense	VP4
UC24	ATCATGGTGTCTATTTCTGC	Antisense	VP3
UG1	TTTGTGTCAGCGTGTAATGA	Sense	VP1
UC1	GAATTCCATGTCAAATCTAGA	Antisense	VP1
Y7	GGGTTTGTGTCAGCCTGTAATGA	Sense	VP3
Q8	AAGAGGTCTCTRTTCCACAT	Antisense	2A
PV1A	TTIAIIGCRTGICCRTTRTT	Antisense	VP1
PV4S	ACITAYAARGAYACIGTICA	Sense	VP1

### Phylogenetic analysis

Sequence data was edited and aligned through Sequencher 4.10.1 (Gene Codes Corporation, USA). MEGA 5 software (www.megasoftware.net) was used to calculate the genetic distances and phylogenetic tree was constructed by the neighbor-joining algorithm [23] with Kimura 2-parameter model using 1000 bootstrap replicate values to correct multiple substitutions rate at a site [24].

### Estimation of the date of initiating OPV dose

We used presumption that nucleotide substitutions occurred in all three poliovirus serotypes at constant rates of 3.2% synonymous substitutions per synonymous site per year (Ks) and 1.1% total substitutions per site per year (Kt) in the 2637-nt P1/capsid region [25]. The approximate “age” (i.e. the time elapsed after the onset of their independent evolution from vaccine) was then estimated from Ks (corrected proportion of synonymous substitutions), Kt (all substitutions per site) values and the date of specimen collection [25].

### Nucleotide sequence accession numbers

5′UTR and P1 coding region sequences of the type 2 polioviruses described in this study were submitted to GenBank library under accession numbers KF322152 (AFG09-1321), KF322153 (AFG10-1094), KF322154 (AFG10-1125), KF322155 (AFG10-1230), KF322156 (AFG10-1924), KF322157 (AFG10-2273), KF322158 (AFG11-066).

## Results

Stool samples from 2646 Afghan children collected from June 2009 to February 2011 were processed for virus isolation and characterization. Out of these, 531 (20%) cases were positive for non-polio enteroviruses, 154 (6%) for polioviruses while 1961 (74%) cases were reported as negative. Among PV positive cases, 109 (71%) were positive for vaccine viruses (Sabin-like PVs) and 45 (29%) for wild type polioviruses on intra-typic differentiation. Sabin like type 1 polioviruses were detected in the highest proportion 51.4% (n = 56) followed by type 3 27.5% (n = 30) and type 2 polioviruses 21.1% (n = 23) respectively. All Sabin-like (SL) viruses were screened for VDPVs using rRT-PCR and 12 positive cases were detected which were sequenced for VP1 gene. Comparative analysis of VP1 sequence of these isolates with respective reference Sabin strain revealed that 6 isolates were Sabin like (SL1 = 2, SL2 = 3 and SL3 = 1) and remaining six were confirmed as VDPV type2.

Among SL2 viruses, only one virus (AFG09/1321) isolated during 2009 from a 12 months old male with quadriplegia from district Bust of Helmand province showed 0.9% nucleotide and 1% amino acid divergence in VP1 region respectively when compared with Reference Sabin 2 strain (GenBank accession No. AY184220.1). It was reported as SL2 because it did not fulfill the old definition of VDPV (≥1% VP1 nucleotide divergence when compared to reference Sabin-2 strain) however the new definition (>0.6% VP1 nucleotide divergence when compared to reference Sabin-2 strain) classified this isolate as cVDPV.

All cases with type2 VDPVs isolation were resident of district Nad Ali of Helmand province and showed residual neurological weakness upon 60 days follow up (Table 2). However, one child AFG10/1125 died within 60 days period (Table 2). Based on their close genetic relatedness and geographical location, these six VDPVs were designated as cVDPVs.

**Table 2:** Demographic description of index case (AFG09/1321) and cVDPVs circulating in Southern Afghanistan.

Sample I.D	Sex	Age (Months)	Symptoms	OPV Doses	Data of Paralysis	Date of Specimen Collection	60 days follow up
AFG09/1321	Male	12	Weakness of all 4 limbs and neck muscles	Unknown	24-Jul-09	29-Jul-09	Weakness limited to left lower limb but spastic rather than flaccid
AFG10/1094	Female	18	Paralysis of left leg with decreased muscle tone and reflexes	0	10-Jun-10	18-Jun-10	Residual weakness
AFG10/1125	Male	27	Paralysis of both leg with decreased muscle tone and reflexes	1	16-Jun-10	23-Jun-10	Died
AFG10/1230	Female	42	Paralysis of right leg with decreased muscle tone and reflexes	15	2-Jul-10	7-Jul-10	Residual weakness
AFG10/1924	Male	11	Paralysis of both leg with decreased muscle tone and reflexes	2	23-Oct-10	29-Oct-10	Residual weakness
AFG10/2273	Male	10	Paralysis of both leg with decreased muscle tone and reflexes	1	18-Dec-10	24-Dec-10	Residual weakness
AFG11/066	Female	36	Paralysis of both leg with decreased muscle tone and reflexes	2	20-Jan-11	24-Jan-11	Residual weakness

These viruses shared 98.2%–97.7% nucleotide and 98.7%–98% amino acid homology in viral capsid protein 1 (VP1) when compared with Sabin type 2 reference strain (Table 3). The overall mean nucleotide and amino acid homologies among these cVDPVs in VP1 region were 99% and 99.6% respectively. Synonymous and non-synonymous mutation detection rates in VP1 region ranged from 4.1% to 6.4% and 0.6% to 1% respectively.

**Table 3:** Characteristics of cVDPVs and index Sabin like type 2 strain (AFG09/1321).

Virus	Serotype Identification by rRT-PCR	Intratypic Differentiation By rRT-PCR	VDPV screening by rRT-PCR	Nucleotide Diversity from Sabin 2 in VP1 (%)	Amino acid Diversity from Sabin 2 in VP1 (%)
AFG09/1321	aP2	bSL	Suspected VDPV	0.9	1
AFG10/1094	aP2	bSL	Suspected VDPV	2.1	1.7
AFG10/1230	aP2	bSL	Suspected VDPV	2	1.3
AFG10/1125	aP2	bSL	Suspected VDPV	1.8	1.3
AFG10/1924	aP2	bSL	Suspected VDPV	2.7	2
AFG10/2273	aP2	bSL	Suspected VDPV	1.8	1.3
AFG11/066	aP2	bSL	Suspected VDPV	2.3	1.7

a P2, Poliovirus type 2.

b SL, Sabin-like.

All cVDPVs showed A→G mutation at nucleotide position 481 in 5′UTR and U→C at nucleotide 2909 in VP1 coding region, leading to amino acid substitution of isoleucine by threonine at codon position 143 (Table 4). Furthermore, five mutations (U_63_→C, U_156_→C, U_398_→C, U_570_→A, and A_736_→G) observed in 5′UTR were common across all cVDPVs (Table 4) while two nucleotide insertions were also observed in AFG10/2273 and AFG11/066 at position 89(C) and 91(G) respectively (Table 4).

**Table 4:** Nucleotide mutations and Amino acid substitutions identified in 5′UTR and P1 Coding region of Index case (1321) and cVDPVs.

Region	Nucleotide									Amino acid								
	Position	Sabin 2	1321	1094	2273	1924	1230	1125	66	Position	Reference Sabin 2	1321	1094	2273	1924	1230	1125	66
**5′**	63	U		C	C	C	C	C	C									
	78	C						A										
	*89	:							G									
	90	A						U	C									
	*91	:			C													
	92	U		C	C	C	C		C									
	97	U		A	A	A	A	A	A									
	156	U	C	C	C	C	C	C	C									
	259	U							C									
	398	U	C	C	C	C	C	C	C									
	403	U						A										
	425	U	C															
	481	A	G	G	G	G	G	G	G									
	517	C			U													
	565	G			A		A		A									
	570	U		A	A	A	A	A	A									
	575	U					C											
	610	U			C				C									
	645	U		C	C	C	C		C									
	657	U				C												
	710	A			G				G									
	716	A				U												
	719	C			U				U									
	727	G						A										
	736	A		G	G	G	G	G	G									
	744	A						C										
**VP4**	753	C	U	U	U	U												
	756	C	U															
	759	A				G												
	780	A					G											
	786	C							U									
	798	C		U		U	U	U	U									
	873	C	U	U	U	U	U	U	U									
	879	A		G	G	G	G		G									
	888	A	U															
	931	A			G					62	Ille			Val				
	945	C			U													
	954	C	U															
**VP2**	963	C				U												
	981	U	C															
	999	G	A															
	1006	C			U													
	1014	U		C		C												
	1071	U							C									
	1122	C		U	U	U	U	U	U									
	1140	G					A											
	1152	A		G	G	G	G	G	G									
	1179	C		U	U		U											
	1290	C	U	U		U	U	U										
	1296	U							A									
	1308	U							C									
	1322	G					U			123	Gly					Val		
	1329	U			C		A			125	Phe					Leu		
	1348	U				C												
	1356	U							C									
	1380	A			G				G									
	1419	A	G															
	1431	U		C	C	C	C	C	C									
	1435	A				G				161	Thr				Ala			
	1437	C	U															
	1440	U		C	C	C	C	C	C									
	1443	U				C												
	1446	C						A										
	1464	A		G		G	G	G										
	1557	U	C															
	1638	C			U													
	1651	C							U									
	1656	G	A	A	A	A	A	A	A									
	1659	A		G			G											
	1674	U						C	C									
	1686	U			C													
	1689	G	A	A	A	A	A	A	A									
	1695	C	U	U	U	U	U	U	U									
	1749	C							U									
	1752	U			C				C									
**VP3**	1833	G					A											
	1866	C		U	U	U	U	U	U									
	1930	C	U							55	Leu	Phe						
	1939	A		U	U	U	U	U	U	58	Thr		Ser	Ser	Ser	Ser	Ser	Ser
	1953	G	A															
	1981	U		C	C	C	C	C	C									
	1990	A	G	G	G	G	G	G	G	75	Thr	Ala	Ala	Ala	Ala	Ala	Ala	Ala
	2010	G		A	A		A	A	A									
	2050	U						C										
	2085	C						U										
	2100	A			G				G									
	2115	U	C															
	2148	C	U															
	2151	A				G												
	2166	U	A															
	2181	A		G	G		G		G									
	2205	A		G	G	G	G	G	G									
	2211	A					G											
	2273	G			C		C		C									
	2274	U							C									
	2286	U			C				C									
	2310	C	U	U	U	U	U	U	U									
	2316	U			C				C									
	2322	A		G	G	G	G	G	G									
	2331	C	U															
	2334	C							U									
	2361	U	C															
	2397	C		U	U	U	U		U									
	2430	U		C	C	C	C	C	C									
	2451	A							U									
	2469	G		A		A												
**VP1**	2499	U							C	10	Val							
	2509	G		A	A	A	A	A	A			Ille	Ille l	Ille	Ille	Ille	Ille	Ille
	2537	U		G	G	G	G	G	G	19	Val		Gly	Gly	Gly	Gly	Gly	Gly
	2543	C	U															
	2547	U				C												
	2553	C							U									
	2555	A	G	G	G	G	G	G	G	25	Asn	Ser	Ser	Ser	Ser	Ser	Ser	Ser
	2558	G							A	26	Ser							Asn
	2568	C	U															
	2580	C	U	U	U	U	U	U	U									
	2611	U		C														
	2622	G		A	A	A	A		A									
	2670	G						A										
	2677	G				A				66	Val				Ile			
	2694	G						A										
	2706	C					U											
	2710	G		A	A					67	Val		Ile	Ile				
	2727	A	G	G	G	G	G	G	G									
	2739	C	U	U	U	U	U	U	U									
	2766	U		C	C	C	C		C									
	2775	A						C										
	2814	U		A		A												
	2829	U							A									
	2832	A				G												
	2838	A		G	G	G	G		G									
	2853	U					C											
	2883	C		U	U	U	U		U									
	2909	U	C	C	C	C	C	C	C	143	Ile	Thr	Thr	Thr	Thr	Thr	Thr	Thr
	2919	U						C										
	2925	A		G	G	G	G	G										
	2928	U		C	C	C	C	C										
	2958	U		C	C	C	C	C	C									
	2992	A				G				171	Asn				Asp			
	3066	A	G															
	3075	C	U															
	3150	U		A	A	A	A		A									
	3138	U	A															
	3195	A				G												
	3219	U	C	C	C	C	C	C	C									
	3234	G				A												
	3237	G				A												
	3261	C							U									
	3358	C						U										
	3459	A	G															

Comparison of complete P1 capsid region with reference Sabin 2 strain showed 1.1%–2.3% nucleotide and 0.5% to 1% amino acid variation. The corrected proportion of synonymous and non-synonymous nucleotide substitutions in P1 capsid were 4.2% to 9.3% and 0.2% to 0.4% respectively. Various silent substitutions found common in all isolates were C_873_→U, G_1656_→A, G_1689_→A, C_1695_→U, C_2310_→U, U_2430_→C, C_2580_→U, A_2727_→G, C_2739_→U, A_2925_→G, U_2928_→C and U_3219_→C (Table 4). Multiple synonymous mutations have also been observed in all studied cVDPVs and index case (AFG09/1321) as well (Table 4). AFG10/1230 had two non-synonymous mutations in VP2 resulting two amino acid changes; Gly_123_→Val and Phe_125_→Leu. Similarly in one isolate (AFG10/1924) threonine was substituted by alanine at codon position 161 in VP2 (Table 4).

Amino acid comparison of VP3 sequences showed the substitution of Thr_75_→Ala in all cVDPVs and AFG09/1321, due to A→G replacement at position 1990, located in the immediate vicinity of antigenic site 3B. Substitution of Thr_58_→Ser in antigenic site 3a (AgS3) was also observed in all studied cVDPV strains; whereas the Leu_55_→Phe change in 3a (AgS3) was only observed in AFG09/1321 (Table 4). Viral capsid protein 4 (VP4) showed 9 nucleotide changes. The VP4 amino acid sequence of all studied strains remained unchanged and only one amino acid substitution Ille_62_→Val has been identified in AFG10/2273.

**Figure 1:**
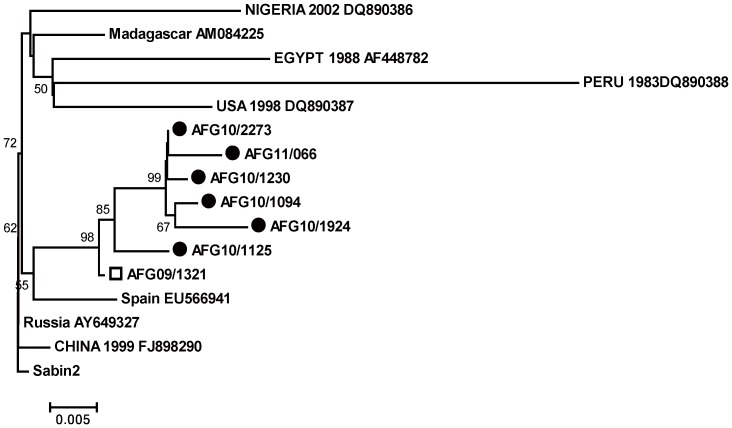
All cVDPVs type2 isolated from Nad Ali district of Helmand province are indicated with round circle filled in black color. Other globally reported cVDPVs type 2 has retrieved from GenBank to reconstruct phylogenetic tree from VP1 coding sequences using neighbor-joining algorithm with Kimura 2-parameter substitution model.

The phylogenetic tree constructed with previously reported cVDPVs from Spain EU566941, Nigeria DQ890386, Russia AY649327, China FJ898290, Madagascar AM084225, USA DQ890387, Peru DQ890388 and Egypt AF448782) revealed that all Afghan cVDPVs belonged to one lineage that originated from AFG09/1321 (Fig. 1). In turn, this isolate appears to have evolved from Sabin 2 vaccine strain and was marked as the index case for the subsequently circulating VDPVs. The approximate time of initiating OPV infection was calculated by Ks and Kt values and date of specimen collection. Analysis showed that index case (AFG09/1321) had 2.5% and 0.8% Ks and Kt value respectively, therefore the estimated time of initial OPV dose administration was determined to be approximately 9 months before the onset of paralysis.

## Discussion

Oral Polio Vaccine has been demonstrated as a powerful tool to stop transmission of PVs for a period spanning over five decades [26]. Although it is considered a safe vaccine and protects individuals through humoral and mucosal immunity, neurovirulent phenotypes known as VDPVs emerge during the course of replication [27], [28]. These VDPVs either emerge during persistent infections in immunodeficient individuals or can evolve during continuous transmission of vaccine viruses in a community where immunization coverage is deficient or patchy [29]. Furthermore, overcrowding, high birth rates, poor sanitation and the absence of wild virus of same serotype as that of VDPVs are major risk factors that facilitate emergence of these viruses and also trigger establishment of numerous independent endemic chains of transmissions [30].

Weak routine immunization and suboptimal campaign quality were the major challenges in southern Afghanistan. Nad Ali is the most populous district of Southern Afghanistan with target population of 99,333 children under 5 years of age. This is one of the districts of Afghanistan that remained endemic for wild polioviruses during 2006 to 2010. Based on AFP surveillance data reports, routine coverage of trivalent oral poliovirus vaccine (tOPV) was 13% during 2008–2009 in South Afghanistan [31]. Therefore during 2008–2009, large scale house to house supplemental immunization campaigns (SIAs) were conducted targeting children <5 years using different formulations (eight immunization rounds with trivalent OPV, eight monovalent type 1 (mOPV1) and four monovalent type 3 OPV (mOPV3), and one round of bivalent OPV containing poliovirus types 1 and 3 (bOPV), however, the vaccination coverage remained less than 50% of the total target population [31], [32]. The vaccination coverage data showed that over 50% of the target children were being missed during the last nine polio vaccination campaigns during 2008–09 [32]. Similarly in February 2010, the entire district could not be covered due to on-going military operation. In later vaccination campaigns during 2010 (March to June) 33–50% targeted children could not be accessed. A number of villages and towns were completely missed during the vaccination campaigns amongst areas where the 6 VDPV2 cases were detected [32]. Therefore suboptimal campaign coverage and inaccessibility to the target population played a major role in the evolution and spread of cVDPVs type 2 in Nad Ali while poor hygienic and sanitation conditions also facilitated viral transmission.

Sequence analysis of VP1 region revealed that the cVDPVs type 2 had evolved from an isolate AFG09/1321, that had been earlier notified as SL2 which did not fulfill the old criterion of VDPVs but in 2011, revised definition of cVDPVs (>0.6% divergent in VP1 region from type2 Sabin reference strain) justified and classified this isolate as cVDPV type 2. This virus carried two signature mutations of virulent phenotype and child also showed cardinal signs of poliomyelitis.

All previously reported mutations responsible for reversion and restoration of neurovirulent properties or elimination of temperature sensitive phenotypes of vaccine derivative were found in the cVDPVs in our study [30], [33]–[36]. However twelve mutations; C_873_→U, G_1656_→A, G_1689_→A, C_1695_→U, C_2310_→U, U_2430_→C, C_2580_→U, A_2727_→G, C_2739_→U, A_2925_→G, U_2928_→C and U_3219_→C were common to all these cVDPVs that have not been previously reported. These mutations may be responsible for increased neurovirulence, enhanced replication capacity and ultimately higher virus titers, prolong period of viral shedding and hence a heightened potential for person to person transmission [37]. Similar observations have previously been reported for revertant viruses [38] and need to be explored further through animal model studies.

The nucleotide data of VP1 gene confirmed the prolonged transmission of OPV derived type2 polioviruses in Nadi Ali district of Afghanistan. The VP1 mutations at aa 143 (Ile_143_→Thr) for type2 vaccine viruses replicating in humans were observed in all studied isolates. VDPVs screening rRT-PCR assay is also based on the presence of these mutations. Furthermore, the amino acid substitution at codon position VP3-75 (Thr_75_→Ser) was also found in all analyzed cVDPVs however, this change relates to results in Enzyme linked Immunosorbant (ELISA) ITD assay that was not conducted in this study [39]. None of the Afghan cVDPV except AFG10/2273 has shown amino acid change at codon position 62 (Ill_62_→Val) that lies within the inner side of the virion and chances for its immune-selection are quite improbable.

In conclusion, our findings provide concrete evidence that circulation of cVDPVs in Southern Afghanistan is result of sub-optimal immunization in the community. The vaccine virus which was progenitor of the reverted strains must have circulated for a long period of time to attain the neurovirulence properties. It is absolutely imperative that more vigorous and determined efforts are required to fill the large immunity gaps through strong routine immunization strategy followed by successive national immunization campaigns. Furthermore enhanced surveillance activities in the border areas of Pakistan and Afghanistan will be instrumental to stop the cross boarder transmission of these viruses and ultimately pave the way towards polio eradication from this region.
